# The Psychological Impact of Confinement Linked to the Coronavirus Epidemic COVID-19 in Algeria

**DOI:** 10.3390/ijerph17103604

**Published:** 2020-05-21

**Authors:** Azzeddine Madani, Saad Eddine Boutebal, Christopher Robin Bryant

**Affiliations:** 1 Faculty of Social and Human Sciences, Khemis-Miliana University, Khemis-Miliana 44225, Algeria; azzeddine.madani@univ-dbkm.dz (A.M.); s.boutebal@univ-dbkm.dz (S.E.B.); 2 Department of Geography, University of Montreal, Montreal, QC H3T 1J4, Canada

**Keywords:** epidemic, psychological impact, coronavirus, stress, anxiety, covid-19, Algeria

## Abstract

The COVID-19 pandemic continues to spread in countries around the world. The impact of this virus is very great on populations following the application of total and partial containment measures. Our study aims to study the psychological impact of total and partial containment applied in Algeria, on 23 March 2020, following the spread of the virus COVID-19 and also studied the habits and behaviors of the Algerian population during this new way of life and this through a cross-sectional survey launched after three days from the start of confinement to quickly assess the impacts over the period from 23 March to 12 April 2020, by an online questionnaire which allowed us to obtain 678 responses from Internet users, who live in confinement in Algeria. According to the gender variable, our sample includes 405 men, or 59.7%, and 273 women, representing 40.3%. The results of the statistical analysis carried out using SPSS version 22.0 software showed that 50.3% of the respondents were in an anxious situation during these first three weeks of confinement. In addition, 48.2% feels stressed, 46.6% of the respondents confirmed to be feeling in a bad mood, and 47.4% do not stop thinking throughout the day about this epidemic and how to protect themselves. In addition, the study shows that 87.9% of the respondents in Algeria found it difficult to follow the confinement instructions. A significant change in the habits of the population was noted especially for the time of going to bed, the time of waking up, and the use of the Internet as well as the hours devoted to daily reading.

## 1. Introduction

The coronavirus pandemic COVID-19 has continued to spread to countries around the world since its first appearance in Wuhan, China, on 31 December 2019 [1] and the declaration of the World Health Organization (WHO), on 26 January 2020, on the high risk of the epidemic in China and worldwide [2]. The number of people tested positive continues to increase, and for 13 April 2020, it had reached 1,773,084 in several countries, which also recorded 111,652 deaths [3]. The daily increase in deaths and confirmed cases has prompted countries to take social distancing measures and other actions related to general and partial containment that are difficult for some countries to enforce. In China, COVID-19 has spread rapidly since its first appearance in Wuhan and has proven to be very dangerous since some affected patients do not have fever and other symptoms which complicate the diagnosis [4].

The report of the National Health Commission of China indicated on 27 January 2020 that people carrying the virus can infect others by respiratory droplets as well as by direct contact [2]. The severity of the disease is summed up in the ability of the virus to spread and the difficulties in identifying those affected to care for them and preventing them from infecting other people [5]. Based on these conclusions, the Chinese government reacted quickly by quarantining a population estimated at 50 million people, and the total closure imposed on Wuhan on 23 January 2020, and this was to set up a total social distancing, which has forced citizens to stay at home to protect them from the virus [6], and prevent the spread of the epidemic to other parts of China. For some, it was a delay in quarantine since it started almost a month later when the virus first appeared [7]. For the other countries, the COVID-19 prevention measures taken in China were the example to follow, but this varied from one country to another because of the means required for their application. The consequences of the total containment of the population certainly has had effects on the economies of countries and on the mental health of people. Increasing psychological effects and fear seems almost to be certain consequences due to quarantine, and anxiety was expected in Wuhan which could increase each time death increased and information about new cases was circulated [8]. 

Algeria, a country much closer to Europe and located in the north of Africa, has been affected by the pandemic. The first case recorded in this country dates from 25 February 2020, when an Italian arrived in Algeria on 17 February and tested positive [9]. The number of infected people reached 1983 (see Table 1), according to the monitoring cell of the Algerian Ministry of Health, Population and Hospital Reform (MSPRH), which also recorded 313 deaths on 13 April 2020 [10]. This pandemic showed the lack of preparation of several European countries and the slow nature of the decision-making processes [11]. This situation was also noted in other countries of Africa including Algeria which took the decision to isolate the region of Blida, the most affected at the start of the epidemic, which was somewhat delayed, and which facilitated the spread of the virus [9]. Other decisions have been taken by the Algerian government, for example also in the context of social distancing through the demobilization of 50% of the workers, the closure of schools, higher education units, and other structures, before extending partial containment from 7:00 p.m. to 7:00 a.m. to all wilayas in the country, while 9 wilayas have had their confinement extended from 3:00 p.m. to 7:00 a.m. The wilaya of Blida, epicenter of the Covid-19 epidemic, in other words the “Wuhan of Algeria”, has also been kept, by government decision, in total confinement imposed since 23 March 2020 given the large number of cases recorded, and which continue at this time, since the strategies of containment and mitigation of the epidemic are based on the nature of the virus and its way of spreading [12]. The Algerian population thus found themselves in a new way of life imposed by total and partial containment, and this social distancing certainly has had an effect on the mental health of patients, healthy people, and medical personnel. It has been shown in previous research, such as that on the psychosocial effects of the Ebola epidemic [13], that the magnitude of the psychosocial effects on individuals and society even at the international level during the onset of an epidemic can be substantial. 

In Algeria, the population has lived since 23 March 2020, about one month, in partial or total confinement. This is a way of life difficult to support for citizens who are not used to staying for several days inside of their homes, which has caused changes in daily habits with negative effects on people. This situation, which has started to become longer, reveals questions related to the impact of confinement on the habits and behaviors of the population in their daily life, and the psychological impact of confinement on Algerian citizens, as well as the impact of socio-demographic variables on the psychological state of citizens during confinement. We also aim to answer the question on the difference in the psychological impact of COVID-19 according to the gender variable, knowing that anxiety and fear in people because of this still unknown disease and the application of containment, considered essential, but which has been causing a slowdown in life and sometimes blockages which can lead to significant psychological and psychiatric disorders marked by depression, panic disorders, and behavioral disorders [14]. Currently, there is no detailed information on the impact of total and partial containment, during this period of the COVID-19 epidemic, on the Algerian population from the psychological point of view, and their behaviors and daily habits. Our study was launched only three days after the establishment of this mode of confinement on Algerian territory; our objectives aim to study the psychological impact of this confinement in the three weeks after its launch following COVID-19, and the design of a questionnaire to measure the psychological impact of containment in Algeria, as well as identifying the main psychological factors of psychological impact. It is also a question of studying the relationship between socio-demographic variables and the psychological impact of containment during the COVID-19 epidemic in Algeria.

This study will allow Algerian health authorities and possibly elsewhere in the countries of the world to better understand the situation, and this in order to take the necessary measures to assist the population during this period of containment which is likely to lengthen as well after this epidemic.

It is signified by containment during coronavirus COVID-19 that the containment procedures were approved by the Algerian state from 23 March 2020 to deal with this epidemic. The psychological impact signifies the various psychological effects of containment in Algeria on the individual, measured in the current study by the sum of the responses to the questionnaire applied in the current study to a sample of respondents. Daily habits represent the totality of practices and behaviors that the individual frequently embodies in his or her daily life such as: washing hands, going to bed and waking up, watching television, and using the internet.

The present study is limited by its subject which studied the psychological impact of containment during the coronavirus COVID-19 in Algeria, by applying for this purpose a questionnaire to measure specific psychological factors; moreover, this study is limited by the number of respondents to the questionnaire, and the short duration of the study from 23 March to 12 April 2020, imposed by the exceptional nature of this confinement and this epidemic.

## 2. Materials and Methods

### 2.1. The Method

In the current study, we used the design of the descriptive survey by an online questionnaire, with the snowball sample 678 due to the conditions of the home confinement accompanying the spread of the coronavirus pandemic; thus, the electronic questionnaire includes items with meanings about the psychological effects of the coronavirus, and, after data collection, these were statistically analyzed by the SPSS program version 22 (SPSS Inc., Chicago, IL, USA). Then, the stage of scientific description came, which is related to the significance of the indicators of the questionnaire items. Therefore, the data were collected using an online questionnaire from different regions of Algeria. Knowing that it was not based on random selection and the study population did not reflect the reality of the general population, we used the statistical approach to describe the results and their analysis was linked through the qualitative indicators that came along with the meanings of the questionnaire items.

### 2.2. Sample and Participants

We adopted a cross-sectional survey to assess the immediate psychological impact on the public during the COVID-19 epidemic using an online questionnaire. With a wide dissemination of the questionnaire with the help of university students, our sampling strategy based on the snowball method is suitable in exceptional cases where it is difficult to communicate with the population to study an urgent health problem related to containment. This method allowed us to obtain 678 responses from Internet users, who are living through this first confinement of the coronavirus epidemic COVID-19 in Algeria. According to the gender variable, our sample includes 405 men, or 59.7%, and 273 women, representing 40.3% of the total sample. For the age variable, 423 of the respondents were aged between 14 and 34 years old or 62.4%, 239 of the respondents were aged between 35 and 54 years old and 35.2%, and 16 of the respondents were aged between 55 and 74 years old or 2.4% of the total number of respondents in the sample. Therefore, we see in the composition of this sample the representation of practically all the age groups of the society concerned by this research issue.

### 2.3. Tools (Online Questionnaire)

The psychological impact of COVID-19 was measured using a global questionnaire measuring the impact of confinement during COVID-19 coronavirus. This questionnaire of 29 items is composed of three subscales: social impact, psychological impact, and impact on mobility. The impact scale of the coronavirus COVID-19 in Algeria was designed on the standards of the Likert scale which includes five response options. 

This means that the average score for the questionnaire items is 3, so a total score greater than three indicates a negative impact on this variable, and when it is less than 3, this means that there is no negative impact within the meaning of this element.

The questionnaire includes in its entirety questions which are concerned with the following sections:

- Social impacts Q1–12
- Psychological impacts Q13–22
- Impacts on Mobility Q23–29

In this study, we focused on the psychological impacts as the only component (psychological impacts subscale). The questionnaire was designed online to facilitate its dissemination and to obtain respondents’ answers immediately.

We focused on the current study on psychological effects and daily habits only, and therefore we clarify the two concepts in that psychological effects designate the emotional changes occurring in the behavior of the individual during interaction with those around him, as measured by individual self-assessment through the items of the questionnaire according to the measurement scale, where this indicates, in quantitative terms, the total score obtained by the respondent to the questionnaire in the dimension related to Psychological effects. The daily social habits are the behaviors which are linked to the process of the daily interactions of the individual in various social situations. This means in our study that the habits linked to the interaction with the coronavirus, such as sleep patterns and hand washing, were measured with direct questions.

The psychological impact section contains 10 items; this means that the total score varies from 50 to 10 with a theoretical average of 30, and it also means that the score which exceeds 30 reflects a negative effect of the psychological factor, and a lower score in relation to 30 expresses the absence of a negative effect of this factor.

### 2.4. The Reliability of the Questionnaire

We used the Alpha-Cronbach coefficient to calculate the reliability on 253 answers, and the results gave a coefficient of 0.799 for the 12 questions related to social impacts, so it is a value which indicates the reliability in the tool of this study. The statistical results also show that the Alpha-Cronbach coefficient, in the 10 questions on psychological impacts, is equal to 0.782, which indicates the reliability of the study tool. For the six questions concerning the impacts on the mobility of the population during this period of total and partial confinement, the statistical results show that the coefficient Alpha-Cronbach is equal to 0.613, a value indicating the reliability of the tool of study. For the entire questionnaire, with its 29 questions on the impacts during the first total and partial containment of the coronavirus epidemic COVID-19, the statistical results show that the Alpha-Cronbach coefficient is equal to 0.831; this value indicates the reliability of the tool used.

### 2.5. The Validity of the Questionnaire

The signified validity of the capacity of the questionnaire to measure what was ready to be actually measured involved the social, psychological, and mobility effects of total and partial confinement on citizens in Algeria. For this, we used the internal validity method of the questionnaire (for 253 individuals), which indicates the correlation between the items of the questionnaire and its overall score. The results are presented in Table 2.

From this table, it is clear that all the Pearson correlation coefficients between the items and the total score of the questionnaire are positive and statistically significant at the level of 0.05 and 0.01. This result means that the questionnaire has a considerable degree of internal validity.

### 2.6. Statistical Analysis

A statistical analysis was performed using SPSS version 22.0 software. Therefore, descriptive statistics were calculated for socio-demographic variables and psychological impact factors. The Pearson correlation coefficient was also used to measure the correlation between various socio-demographic variables and the psychological impact of coronavirus containment COVID-19. In addition, the multiple regression analysis method was used to measure the effect of socio-demographic variables on the psychological effects of confinement, and the *t*-test was also used to study differences in the psychological impact of coronavirus confinement between men and women.

## 3. Results

### 3.1. Habits of Daily Life during the Coronavirus Epidemic COVID-19 in Algeria

The results obtained show the impacts of confinement during the first total and partial confinement operations of the coronavirus epidemic COVID-19 in Algeria on certain habits of the daily life of citizens, where we note a high rate of hand washing during the day, since 51.77% of the study sample reported washing their hands up to 10 times a day, and 36.73% of the population washed their hands between 10 and 20 times a day. On the other hand, the rest of the sample 11.5% paid special and somewhat exaggerated attention to hand washing between 20 and 40 times a day (see Table 3). We also note that 83.63% of those questioned confirm that they sleep late between midnight and 3:00 a.m., and that 12.09% of people go to bed in the regular period between 8:00 p.m. and 11:00 p.m., while the remaining 4.28% would sleep between 4:00 a.m. and 7:00 a.m. the next day. Thus, we can deduce the considerable impact of the first period of total and partial confinement following the coronavirus epidemic COVID-19, on the hour of going to sleep, considered to be very late.

Regarding waking up time, the survey results show that 45.72% of the study sample confirmed that they woke up between 10:00 a.m. and 12:00 p.m., and that 38.5% said they woke up between 7:00 a.m. and 9:00 a.m., while 3.83% of the respondents woke up between 1:00 p.m. and 3:00 p.m. For the time spent watching TV, the survey results showed that 45.28% of respondents spend up to 5 h watching TV every day, and 33.18% of citizens confirmed that they watch TV for about 10 h every day. On the other hand, we find that 21.54% of the respondents watch television programs daily for around 15 h. Knowing that television is an important means of passing time during the period of the coronavirus pandemic COVID-19 through the many programs and TV channels, the survey results shown in Table 2 also show that 36.72% of respondents, during this period of total and partial containment linked to the COVID-19 pandemic, say that they do not read books, and 34.47% devote one hour a day to reading books, and 13.87% of the respondents read books for 2 h a day. On the other hand, 14.74% of the population prefer to read between 3 and more than 5 h. What is noticed here is poor reading, but perhaps reading books is compensated by electronic reading on smart-phones and computers, and this is shown later.

The respondents confirmed that they use the Internet for several hours a day, so 37.9% of them spend between 10 and 15 h a day, and 33.93% spend between 5 and 10 h maximum a day surfing the Internet. As for the remaining proportion, it uses less internet, 0 to 5 h a day. Indeed, there is a strong dependence on the internet and related devices that allow time to pass during the period of the pandemic.

Finally, we find that 51.92% of respondents are interested in the content of social networks (Facebook and Twitter), and that of YouTube, while 29.94% prefer reading including scientific research by electronic means. In addition, 18.4% of respondents prefer to follow new local and international news related to COVID-19 and other areas on the internet (see Table 3).

The Table 4 above shows the correlation matrix between certain study variables; we note the presence of a statistically significant negative correlation between the variables age and waking time, which means that young people get up later by comparison with the people in the older age categories (*r* = −0.216 **); as shown in the table, there is a statistically significant positive correlation between the variable psychological impacts and waking time (*r* = 0.145 ** ), which means that the increase in sleep time and the delay in getting up are linked to the increase in the level of psychological effects during the first confinement of the coronavirus epidemic COVID-19.

### 3.2. The Psychological Impact of Confinement during the Coronavirus Epidemic COVID-19 in Algeria

The results in Table 5 above show the relative levels and weights of the psychological impact factors of the coronavirus COVID-19 in Algeria during the first total and partial confinement, knowing that the value 3 signifies the theoretical average. According to the respondents, we find that the item related to the difficulty of voluntary engagement in home confinement is ranked first in the psychological factors, with a mean of 4.11, and this signifies a lack of social consciousness and previous experiences in behavior during an epidemic. The rapid spread of the epidemic may not have left the time necessary for better awareness among citizens of the seriousness of the coronavirus COVID-19 and the usefulness of home confinement as the sole means of current prevention.

In second position for the psychological factors, we find anxiety with a mean of 3.22 where the respondents confirmed their feelings of anxiety during the confinement period, perhaps because there are difficulties in accepting confinement itself, or difficulty organizing family life inside the house. In addition, anxiety is strongly present in the event of an epidemic among fragile personalities and contributes to the deterioration of the psychological state of the individual, which affects his or her daily interactions and even his or her physical functions.

The state of psychological stress is the third psychological factor affecting individuals during the coronavirus pandemic COVID-19 in Algeria during this period from 23 March to 12 April 2020, and this is confirmed by the respondents with a mean of 3.18. Admittedly, the spread of the epidemic and the obligation of confinement at home on the one hand, and the difficulty of coping with it, on the other hand, put the individual in a state of psychological stress, especially with the transformation of daily life into a boring daily routine. The fourth psychological factor (see Table 5) affecting individuals during confinement is a mood fluctuation with a mean of 3.17, which reflects the entry of the individual into a state of being emotionally unstable, and which negatively affects him or her and the family environment, not only because of the feeling of limited living space, but also because of a feeling of fear of the pandemic and its various repercussions.

The fifth psychological factor represents dependence on thinking throughout the day about the subject of the epidemic, and this is confirmed by the respondents with a mean of 3.11. This indicates an addiction of thinking about the coronavirus COVID-19, its dangers, and its consequences in an exaggerated way, which leads to psychological, moral, and physical fatigue, and especially in relation to the monitoring of new information which is sometimes incorrect about coronavirus COVID-19. Regarding the rest of the items and psychological factors, the current study did not show any negative effect on the sample of our research, since its arithmetic mean is lower than the theoretical mean 3.

### 3.3. Psychological Impact and Socio-Demographic Variables

According to Table 6, in the multiple regression analysis, it is shown that the variables of sex, age, and family situation were significantly associated (*AR*^2^ = 0.019) with the scores of the psychological impact subscale. Through this table, we find that gender was significantly associated with psychology impact scores (B = 0.112, 95% CI). In addition, age was significantly associated with lower psychology impact scores (B = −0.081, 95% CI). In addition, the family situation was significantly associated with psychology impact scores (B = 0.079, 95% CI).

### 3.4. Differences in the Psychological Impact of the Coronavirus COVID-19 according to the Gender Variable

Table 7 below presents the study of the differences in psychological impact between men and women during the first confinement of the coronavirus epidemic COVID-19 in Algeria; the results show the existence of statistically significant differences in favor of women (M = 31.35) compared to men (M = 29.63) in the psychological impact scale.

This result means that the female population is more affected by coronavirus COVID-19 than men, and, to determine the details, we return to the differences in the statistically significant items. Indeed, women were more delusional than men, more eager to wash their hands too much, presenting more emotional stress, fear and an unstable mood, and they were more unreal optimists that they would never be infected by the coronavirus COVID-19.

## 4. Discussion

The results were discussed according to the structure of presenting the data by linking them to previous studies according to what we had, especially since the problem is recent.

The results obtained to see the changes in behavior and habits as well as the psychological impacts on the Algerian population during the first three weeks (from 23 March to 12 April 2020) of the total and partial confinement applied by the Algerian government show that the difficulty of voluntary engagement in home confinement is ranked first in terms of psychological factors, especially since 87.9% of the respondents have difficulty applying the confinement instructions. Our field observations confirm that some people often leave their homes and do not follow or have difficulty applying the instructions for containment. The lack of awareness through the dissemination of specialized information affects the population, which remains worried in the absence of reliable information, while previous research has revealed the presence of a wide range of psychosocial impacts on people at the individual, community, and international levels during the spread of the epidemic [6]. It is also possible that the rapid spread of the epidemic has not left the time necessary for better awareness among citizens of the usefulness of levels of home confinement as the only means of prevention. Among other things, 50.3% of respondents indicated that they are in an anxious situation for various reasons related to a new organization of daily life, in addition to the measure of confinement or quarantine that shows that the authorities consider the serious situation and its risk of worsening [8], and this worries the population; and the rapid increase in anxiety in people is linked to the lack of information on the disease and the preventive measures that produce a blockage in daily life [14].

It is also found that 48.2% of respondents experience stress during the period of total and partial containment, and certainly people are well informed that COVID-19 threatens the lives of people and that there is no treatment in this current period, which has triggered a wide variety of psychological problems [16] in the population. In addition, there is the transformation of everyday life into very limited actions which in time becomes very boring. In addition, 46.6% of the surveyed population confirmed feeling in a bad mood during this first period of confinement, which means that the individual is in an unstable emotional state which negatively affects him or her as well as the family environment. In addition, 47.4% of respondents continue to reflect throughout the day on this epidemic and on the ways to protect themselves, and this dependence in an exaggerated way leads to psychological, moral, and physical fatigue.

The Chinese government has improved public awareness of prevention measures, and psychologists and psychiatrists use the internet and social media to share strategies for managing psychological stress [17]. The results of this survey showed that women are the most affected compared to men by the impacts of confinement linked to COVID-19. Therefore, women prefer to wash their hands several times, are more stressed and manifest more fear and instability of mood while they are also more unreal optimists that they would never be infected by the coronavirus COVID-19.

The change in population behavior during confinement also affects psychological and physical health, so, during this 3-week period 51.77% of respondents indicated that they wash their hands up to 10 times a day, and 36.73% do it between 10 and 20 times a day. On the other hand, 11.5% of the respondents exaggerate in terms of hand washing and do it between 20 and 40 times a day; this category of people either move frequently outside, in regions of partial containment and know perfectly the hygienic rules which pushes them to react like this. Either he or she lives in the region where the confinement is total which forces them, for fear, to wash his or her hands regularly even at home.

Note that 83.63% of those questioned sleep late between midnight and 3:00 a.m. and that only 12.09% go to bed in normal h between 8:00 p.m. and 11:00 p.m. On the other hand, 4.28% of respondents say that they will go to bed between 4:00 a.m. and 7:00 a.m., which shows that confinement has changed their habits, since schools are closed, and life is slowing down. In this same context, 45.72% of respondents woke up in the morning between 10:00 a.m. and noon and 38.5% indicated that they woke up between 7:00 a.m. and 9:00 a.m. 3.83% of respondents wake up between 1:00 p.m. and 3:00 p.m.; this category represents that of young people who stay connected to the internet for a long time. These changes in the time to go to bed and wake up are a sign of an increase in the level of psychological effects during this first confinement. In addition, there are many hours spent watching television since 21.54% of respondents say they spend 10 to 15 h watching television programs daily, and 33.18% spend between 5 to 10 h watching television daily. The population is also trying to follow the information associated with the epidemic COVID-19 on the various international TV channels since the gravity of the epidemic has not been widely broadcast or recognized, which has delayed protection measures and also containment [7], which pushes citizens to search for information themselves.

We also find that 45.28% stay up to 5 h in front of the television, which becomes an essential means to follow the new information on the epidemic and the measures taken by many countries which continue to make efforts to minimize the contacts between humans and guarantee good protection for the population [18], especially since it is always difficult to fight against COVID-19 of unknown origin and mysterious biological characteristics with a long period of incubation [19].

Reading books, during the period of total and partial confinement in Algeria, does not interest 36.72% of respondents, while 34.47% devote one hour per day to reading and 14.74% of the surveyed population prefer to read books for between 3 h and more than 5 h a day. Confined people do not pay much attention to reading since it is certainly linked to reading on digital media via the Internet for many hours.

Thus, 37.9% of respondents say that they devote to the internet between 10 and 15 h per day, and 33.93% remain connected to the internet between 5 and 10 h per day, which constitutes a strong dependence on the internet and its services during the containment period. Social networks (Facebook, Twitter, and YouTube) attract the attention of 51.92% of respondents. On the other hand, 29.94% prefer reading and scientific research on the internet; 18.4% of the surveyed population opt to follow new local, national and international information related to COVID-19 and other subjects, since it would not be surprising that one day, in the near future, broader containment measures will be required to protect against this pandemic [20].

For this purpose, it is necessary to use the different means of information and communication so that psychologists increasingly approach the confined population in need of psychological help. The dissemination of information related to COVID-19 must be carried out with complete transparency and by specialized scientific journalists capable of disseminating most of the information with great precision. Identifying confined people through a platform and remote assistance will make it possible to quickly get closer to people in urgent need of psychological support.

## 5. Conclusions

The COVID-19 pandemic continues to spread in countries all over the world. The number of people affected and deaths is increasing every day. The impacts of this are very big on the populations following the application of total and partial containment measures. Our study evaluated the psychological impact of total and partial confinement applied in Algeria, on 23 March 2020, following the spread of the virus COVID-19 and we also studied the habits and behaviors of the Algerian population during this new mode of life, and this through an investigation launched after three days of the start of confinement to quickly assess the impacts over the period from 23 March to 12 April 2020, by an online questionnaire.

The results showed that 50.3% of respondents were in an anxious state during these first three weeks of confinement. In addition, 48.2% feel stressed, 46.6% of the respondents confirmed feeling in a bad mood, and 47.4 % do not stop thinking throughout the day about this epidemic and how to protect themselves.

In addition, the study shows that 87.9% of respondents in Algeria found it difficult to follow the instructions for full and partial containment. A significant change in the habits of the confined population, especially about going to bed and waking up time, is observed, which shows the increase in the level of psychological effects. 

Note also that changes in internet use and daily reading are seen in the results of this study. Among others, the limitations of this study are linked to the sampling strategy, the number of respondents, and the short duration of the study. Thus, this will not make it possible to generalize these results over the entire population. However, these results can help the health authorities and other services concerned by the epidemic COVID-19 in the procedures for taking charge of the population during this period of confinement, which is likely to lengthen further, knowing that the psychological aspect, which influences behavior, is very important to fight against the coronavirus. It is also necessary to regularly monitor the change in daily habits since it indicates the level of awareness of citizens about health protection. In this type of situation, psychological support must be provided remotely to families and individuals to alleviate their suffering and encourage them to stay at home during the confinement period and to respect the habits of prevention against the coronavirus. The current study can be developed to study the effect of confinement on personality characteristics, quality of life, and link them to behavioral habits to be more preventive.

## Figures and Tables

**Table 1:** Evolution of the COVID-19 epidemic in Algeria in the period from 23 March to 13 April 2020.

Date	New Infected Per Day	Total Infected	New Deaths/Day	Total Deaths
23 March	29	230	0	17
27 March	42	409	1	26
30 March	73	584	4	35
3 April	185	1171	22	105
4 April	80	1251	25	130
5 April	69	1320	22	152
6 April	103	1423	21	173
7 April	45	1468	20	193
8 April	104	1572	12	205
9 April	94	1666	30	235
10 April	95	1761	21	256
11 April	64	1825	19	275
12 April	89	1914	18	293
13 April	69	1983	20	313

Source: the authors based on the data [15].

**Table 2:** Internal validity of the questionnaire (253 respondents).

Pearson Correlation					
Q1	Q2	Q3	Q4	Q5	Q6
0.525 **	0.205 **	0.206 **	0.282 **	0.467 **	0.166 **
Q7	Q8	Q9	Q10	Q11	Q12
0.325 **	0.465 **	0.391 **	0.133 *	0.233 **	0.135 *
Q13	Q14	Q15	Q16	Q17	Q18
0.607 **	0.678 **	0.703 **	0.558 **	0.478 **	0.165 **
Q19	Q20	Q21	Q22	Q23	Q24
0.516 **	0.592 **	0.401 **	0.520 **	0.182 **	0.166 **
Q25	Q26	Q27	Q28	Q29	
0.147 *	0.207 **	0.163 **	0.525 **	0.328 **	

* The correlation is significant at the 0.05 level; ** The correlation is significant at the 0.01 level. Source: Authors using SPSS version 22.0.

**Table 3:** Descriptive statistics of the variables for daily living habits during the first confinement of the coronavirus pandemic COVID-19 in Algeria.

Variables	The Interval	Frequency	Percentage
Wash your hands	0–10	351	51.77
	10–20	249	36.73
	20–30	57	08.41
	30–40	21	03.09
Time to go to bed	8 p.m.–11 p.m.	82	12.09
	12 a.m.–3 a.m.	567	83.63
	4 a.m.–7 a.m.	29	04.28
Wake up time	4 a.m.–6 a.m.	81	11.95
	7 a.m.–9 a.m.	261	38.50
	10 a.m.–12 p.m.	310	45.72
	1 p.m.–3 p.m.	26	03.83
Watch the television	0 h–5 h	307	45.28
	5h–10 h	225	33.18
	10 h–15 h	146	21.54
Reading	0 h	249	36.72
	1 h	235	34.67
	2 h	94	13.87
	3 h	44	06.48
	4 h	19	02.80
	5 h	15	02.22
	More than 5 h	22	03.24
Internet usage	0 h–5 h	143	21.10
	5 h–10 h	230	33.93
	10 h–15 h	257	37.90
	15 h and more	48	07.07
How to use the Internet?	Facebook, Twitter, You Tube	352	51.92
	Reading and scientific research	203	29.94
	Track new information	123	18.14

Source: Authors using SPSS version 22.0.

**Table 4:** The correlation matrix between the age and time of waking up and the psychological impact during the first confinement of the coronavirus pandemic COVID-19 in Algeria.

The Variables	Age	Wake up Time	Psychological Impact
Age	1	−0.216 **	−0.070
Wake up time	−0.216 **	1	0.145 **
Psychological impact	−0.070	0.145 **	1

** The correlation is significant at the 0.01 level (bilateral). Source: Authors using SPSS version 22.0.

**Table 5:** The relative weight of the items in the questionnaire on the psychological impact of the. coronavirus epidemic COVID-19 in Algeria during the first total and partial confinement (*n* = 678).

Items	*n* = 678			Mean	Relative Weight	Rating
	Responses	*n*	%			
I imagine I am infected with the coronavirus COVID-19	Not agree at all	161	23.7	2.67	53.40	08
	Disagree	180	26.5			
	Neutral	110	16.2			
	Agreed	178	26.3			
	Totally agree	49	7.2			
I repeat hand washing exaggeratedly	Not agree at all	91	13.4	2.91	58.20	06
	Disagree	217	32.0			
	Neutral	126	18.6			
	Agreed	152	22.4			
	Totally agree	92	13.6			
I feel stress during home containment	Not agree at all	82	12.1	3.18	63.60	03
	Disagree	162	23.9			
	Neutral	107	15.8			
	Agreed	207	30.5			
	Totally agree	120	17.7			
I feel anxious during home containment	Not agree at all	64	9.4	3.22	64.40	02
	Disagree	180	26.5			
	Neutral	93	13.7			
	Agreed	228	33.6			
	Totally agree	113	16.7			
I feel fear during home containment	Not agree at all	119	17.6	2.63	52.60	09
	Disagree	240	35.4			
	Neutral	143	21.1			
	Agreed	122	18.0			
	Totally agree	54	8.0			
Difficult to accept home containment instructions	Not agree at all	10	1.5	4.17	83.40	01
	Disagree	15	2.2			
	Neutral	57	8.4			
	Agreed	363	53.5			
	Totally agree	233	34.4			
I feel angry for the most trivial reasons during home containment	Not agree at all	185	27.3	2.39	47.80	10
	Disagree	234	34.5			
	Neutral	115	17.0			
	Agreed	99	14.6			
	Totally agree	45	6.6			
I think I will never be infected with the coronavirus COVID-19	Not agree at all	69	10.2	2.89	57.80	07
	Disagree	165	24.3			
	Neutral	274	40.4			
	Agreed	113	16.7			
	Totally agree	57	8.4			
I feel in a bad mood during home containment	Not agree at all	72	10.6	3.17	63.40	04
	Disagree	140	20.6			
	Neutral	150	22.1			
	Agreed	231	34.1			
	Totally agree	85	12.5			
I think about the coronavirus all day COVID-19	Not agree at all	88	13.0	3.11	62.20	05
	Disagree	173	25.5			
	Neutral	95	14.0			
	Agreed	222	32.7			
	Totally agree	100	14.7			

Source: Authors using SPSS version 22.0.

**Table 6:** Association between demographic variables and the psychological impact of the coronavirus COVID-19 in Algeria during the first containment of the epidemic (*n* = 678).

Variables	Categories	*n %*	R-Squared *R*^2^	Adjusted R-Squared *AR*^2^	B (95%)
Sex	Male	405 (59.7)	0.023	0.019	0.112 *
	Female	273 (40.3)			(0.521 to 2.651)
Age	(14–34)	423 (62.4)			−0.081 *
	(34–54)	239 (35.2)			(−0.117 to −0.001)
	(54–74)	16 (2,4)			
Family Situation	Single	408 (60.2)			0.079 *
	Married	270 (39.8)			(−0.007 to 2.210)

* Significant (0.05); Source: Authors using SPSS version 22.0.

**Table 7:** The different psychological impacts of the coronavirus COVID-19 between men and women in Algeria during the first containment of the epidemic (*n* = 678).

Items	Men (405)		Women (273)		T	Sig
	Mean	Standard Deviation	Mean	Standard Deviation		
I imagine I am infected with the coronavirus COVID-19	2.55	1.237	2.84	1.344	−2.766	0.006
I repeat hand washing exaggeratedly	2.80	1.257	3.07	1.279	−2.682	0.007
I feel stress during home containment	3.10	1.225	3.30	1.413	−1.886	0.050
I feel anxious during home containment	3.15	1.203	3.32	1.349	−1.649	0.100
I feel fear during home containment	2.41	1.065	2.96	1.296	−5.760	0.001
Difficult to accept home containment instructions	4.13	0.792	4.23	0.779	−1.721	0.086
I feel angry for the most trivial reasons during home containment.	2.35	1.165	2.44	1.285	−0.909	0.364
I think I will never be infected with the coronavirus COVID-19	2.99	1.064	2.73	1.060	3.126	0.002
I feel in a bad mood during home containment	3.08	1.136	3.30	1.286	−2.289	0.022
I think about the coronavirus all day COVID-19	3.06	1.306	3.18	1.283	−1.123	0.262
Total	29.6346	6.35733	31.3590	7.59373	−3.200	0.001

Source: Authors using SPSS version 22.0.
